# Comparative Analysis of Chemical Profiles and Biological Activities of Essential Oils Derived from *Torreya grandis* Arils and Leaves: In Vitro and In Silico Studies

**DOI:** 10.3390/plants13182640

**Published:** 2024-09-21

**Authors:** Pengfei Deng, Huiling Wang, Xiaoniu Xu

**Affiliations:** 1 School of Forestry & Landscape Architecture, Anhui Agricultural University, Hefei 230036, China; pengfei_deng@stu.ahau.edu.cn (P.D.); huilingwang@ahjzu.edu.cn (H.W.); 2 Anhui Provincial Key Laboratory of Forest Resources and Silviculture, Anhui Agricultural University, Hefei 230036, China; 3 School of Architecture & Planning, Anhui Jianzhu University, Hefei 230022, China

**Keywords:** *Torreya grandis*, essential oil, B16/F10 melanoma cells, RAW 264.7 macrophages, network pharmacology, molecular docking

## Abstract

*Torreya grandis* (*T. grandis*, Taxaceae) is a well-known nut tree species. Its fruit aril and leaves possess a unique aroma, making it an ideal natural raw material for extracting essential oils (EOs). This study aims to comprehensively compare the composition, biological activities, and pharmacological mechanism of EOs extracted from the arils (AEO) and leaves (LEO) of *T. grandis*. The results revealed that the chemical composition of the two EOs was highly consistent, with *α*-pinene and D-limonene as the main components. Both EOs significantly reduced cellular melanin production and inhibited tyrosinase activity in *α*-MSH-stimulated B16 cells (*p* < 0.05). AEO and LEO suppressed inflammatory responses in LPS-stimulated RAW 264.7 macrophages, significantly inhibiting cellular NO production and proinflammatory cytokines such as TNF-*α* and IL-6 (*p* < 0.05). A network pharmacology analysis reveals that AEO and LEO share similar molecular mechanisms and pharmacological pathways for treating skin pigmentation and inflammation. Regulating inflammatory cytokines may be a critical pathway for AEO and LEO in treating skin pigmentation. These findings suggest that AEO and LEO have potential for cosmetic applications. The leaves of *T. grandis* could be a valuable source of supplementary materials for producing *T. grandis* aril EO.

## 1. Introduction

Chinese Torreya (*Torreya grandis* cv. ‘Merrillii’; *T. grandis*) is the sole exceptional variety within the *Torreya* genus, belonging to the Taxaceae family, and has been selectively cultivated through grafting [1]. *T. grandis* is primarily distributed in subtropical coniferous and broad-leaved mixed forest zones, covering regions in East China, South China, and Southwest China. This unique and renowned nut tree boasts a substantial cultivation history in China, serving various economic purposes, ranging from food, medicinal uses, timber production, edible oil extraction, and environmental benefits [2]. The Torreya fruit has three primary components: the aril, outer seed coat, and edible kernel. Despite the aril comprising 50% to 60% of the fresh fruit mass, Torreya fruit is often discarded through landfilling, incineration, or composting [3]. Essential oil (EO) derived from the aril of *T. grandis* (AEO) represents a by-product of Torreya nut production aimed at increasing its industrial value and mitigating environmental pollution. *α*-pinene and D-limonene emerge as predominant constituents of AEO when extracted through hydro-distillation, constituting approximately 50% [4]. AEO has been reported to possess multiple biological activities, such as antioxidant [5], antibacterial [3], antiseptic [6], antiparasitic [4], and tyrosinase inhibition properties [7], establishing it as a readily available and cost-effective source of natural antioxidants for the cosmetics industry.

However, seasonal limitations present a significant challenge in AEO production. While there is an excess of aril waste during the October harvesting season, there is no aril supply for production during other months, representing a substantial hurdle for the expansion of AEO production. One potential solution to this issue is extracting EO from the leaves of *T. grandis*. Its leaves are characterized by fragrance and also contain EO. As the Torreya tree is an evergreen species, leaf extraction is not subject to seasonal constraints. This approach could offer a more consistent and sustainable source of Torreya EO year-round, supporting the growth of the Torreya EO industry. While Saeed et al. (2010) indicated that the organic-phase extract of *T. grandis* leaves contains alkaloids, flavonoids, tannins, terpenoids, and saponins, which exhibit analgesic and anti-inflammatory properties [8], the composition and biological activities of EO extracted from *T. grandis* leaves (LEO) have not been extensively documented, especially in comparison to the aril-sourced EO. Further exploration, especially regarding the functional agents of cosmetics, would improve our understanding of LEO and its comparative advantages over AEO.

Skin lightening has emerged as a significant concern in cosmetics, particularly in Asia. Hyperpigmentation, such as that seen in freckles, solar lentigines, melasma, aging, and melanoma, poses aesthetic and dermatological challenges [9]. Tyrosinase, the crucial enzyme catalyzing the rate-limiting step in melanin biosynthesis, triggers the hydroxylation of L-tyrosine to 3,4-dihydroxyphenyl-L-alanine (L-DOPA), followed by the oxidation of L-DOPA to dopaquinone. The oxidative polymerization of various dopaquinone derivatives leads to the formation of melanin [10,11]. Functional hypopigmentation agents may decrease melanogenesis by reducing cellular tyrosinase activity. Melanin biosynthesis is triggered by a variety of stimuli, with UV-induced *α*-melanocyte-stimulating hormone (*α*-MSH) being the principal stimulant of melanogenesis in melanocytes [11]. Oxidative stress can also lead to melanogenesis. Other reactive oxygen species (ROS) and hydrogen peroxide (H_2_O_2_) may expose melanocytes to high-grade oxidative stress, thereby modulating melanogenesis [12]. Antioxidants are recognized for their anti-melanogenic properties, as they can scavenge free radicals and interact with melanogenic intermediates [13]. Moreover, post-inflammation may lead to abnormal hyperpigmentation. Research has shown that cytokines and chemokines released during inflammation can stimulate the proliferation of melanocytes, the production of melanin, and their transfer to neighboring keratinocytes [14,15]. Consequently, boosting antioxidative and, in particular, anti-inflammatory capacities represents a promising approach for creating skin-lightening agents. EOs are a beneficial natural source of tyrosinase inhibitors, antioxidants, and anti-inflammatory agents [15,16]. Various aromatic compounds in EOs, including terpenes and terpenoids, have been shown to play a significant role in biological activities such as skin whitening, antioxidation, and anti-inflammatory effects [15].

In the present study, AEO and LEO were extracted via hydro-distillation, and their chemical constituents were identified using gas chromatography–mass spectrometry (GC-MS). The inhibitory effects of AEO and LEO on lipid peroxidation were evaluated using the *β*-carotene–linoleic acid bleaching inhibition assay. Additionally, the study developed models of α-MSH-stimulated B16/F10 melanoma cells and LPS-stimulated RAW264.7 cells to assess the in vitro melanogenesis inhibition and anti-inflammatory activities of AEO and LEO, respectively. The primary objectives of this study were to (1) comprehensively compare the extraction yield and composition of AEO and LEO obtained using the same method; (2) investigate the efficacy and differences of AEO and LEO in melanogenesis inhibition, antioxidation, and anti-inflammatory effects. Network pharmacology, a contemporary analytical technique that integrates high-throughput omics data with network databases, facilitates the direct identification of disease targets for complex drugs and predicts the mechanisms and pathways that govern the multifaceted actions of these drugs [17]. The study further employed network pharmacology to investigate the protective effects and pharmacological mechanism of AEO and LEO in melanogenesis inhibition and anti-inflammatory activities, with the additional aim of (3) comparing the potential molecular mechanisms of AEO and LEO in melanin and inflammation inhibition. Overall, this research seeks to elucidate the potential applications of *T. grandis* leaves as an auxiliary resource in producing *T. grandis* aril EO.

## 2. Results

### 2.1. Chemical Compositions and Yield of EOs from Aril and Leaves of T. grandis

**Figure 1:**
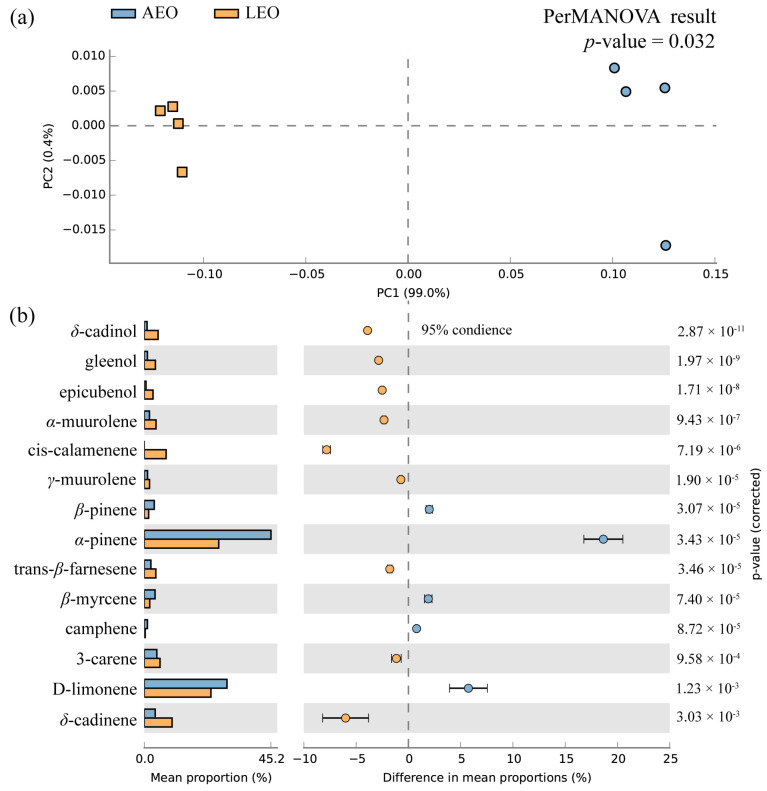
(**a**) Principal component analysis of components isolated from the samples of *Torreya grandis* aril (AEO) and leaves (LEO). (**b**) The difference in the percentages of the major components of AEO and LEO.

A comparative analysis of the chemical compositions and extraction yields for AEO and LEO is depicted in Table 1. The yields of AEO and LEO differed significantly (*p* < 0.05), with AEO exhibiting the highest yield at 2.04 mg/g, almost five times greater than that of LEO at 0.49 mg/g. Upon comparing their mass spectra and retention indices with the NIST library, 42 and 47 compounds were identified in AEO and LEO, respectively, representing 96.81% and 97.64% of their respective compositions. The Venn diagram in Figure S1 illustrates the presence of 39 common compounds in both EOs, accounting for 78% of the total compounds, suggesting that the two EOs share similarities in compound composition. Monoterpenes dominated the composition of the two EOs. However, AEO and LEO differ in their relative abundance of compounds. Compared with AEO, LEO displays a lower relative content of monoterpenes but a higher relative content of sesquiterpenes, oxygenated monoterpenes, and oxygenated sesquiterpenes. The PCA plot (Figure 1a) reveals a significant separation between the two EOs. The PerMANOVA test, utilizing Bray–Curtis distance measures, demonstrates a statistically significant difference in the chemical composition distribution (*p* < 0.05, permutations = 999; Figure 1a) between AEO and LEO. The volcano plot (Figure S2) details the differences in compound content between AEO and LEO, with 27 compounds up-regulated (*p* < 0.05 and FC > 1.5) and 13 compounds down-regulated (*p* < 0.05 and FC > 1.5) in LEO compared to AEO.

The predominant components identified in AEO and LEO are depicted in Figure 1b. The top ten most abundant compounds in AEO were *α*-pinene (39.15 ± 1.23%), D-limonene (25.57 ± 0.53%), 3-carene (3.89 ± 0.16%), *δ*-cadinene (3.42 ± 1.24%), *β*-myrcene (3.33 ± 0.17%), *β*-pinene (3.10 ± 0.05%), trans-*β*-farnesene (2.05 ± 0.10%), *α*-muurolene (1.61 ± 0.08%), *γ*-muurolene (1.01 ± 0.05%), and camphene (0.99 ± 0.05%). For LEO, the primary components were *α*-pinene (21.26 ± 0.27%), D-limonene (19.04 ± 1.04%), *δ*-cadinene (7.95 ± 0.08%), cis-calamenene (6.25 ± 0.16%), 3-carene (4.51 ± 0.21%), *δ*-cadinol (3.94 ± 0.04%), *α*-muurolene (3.36 ± 0.04%), trans-*β*-farnesene (3.32 ± 0.03%), gleenol (3.19 ± 0.05%), and epicubenol (2.50 ± 0.04%). In comparison with AEO, LEO demonstrated a significant increase in the relative abundance of *δ*-cadinene, cis-calamenene, 3-carene, *δ*-cadinol, *α*-muurolene, trans-*β*-farnesene, gleenol, epicubenol, *δ*-cadinene, and *γ*-muurolene, while exhibiting a significant decrease in the relative abundance of *α*-pinene, D-limonene, *β*-myrcene, *β*-pinene, and camphene.

### 2.2. Biological Activity Comparison of EOs

#### 2.2.1. In Vitro Anti-Melanogenic Activity

The impacts of AEO and LEO on B16/F10 melanoma cells following stimulation with *α*-MSH were explored. A standard CCK-8 assay was conducted to determine the highest inhibitory concentration of the EOs for further analysis. The assay covered a range of concentrations (10, 20, 30, 40, and 50 µg/mL) with the results shown in Figure S3. The findings indicated that the cell viability of AEO and LEO did not significantly differ at concentrations below 40 µg/mL compared with the control group. Accordingly, EO concentrations between 10 and 40 µg/mL were selected for further experiments.

**Figure 2:**
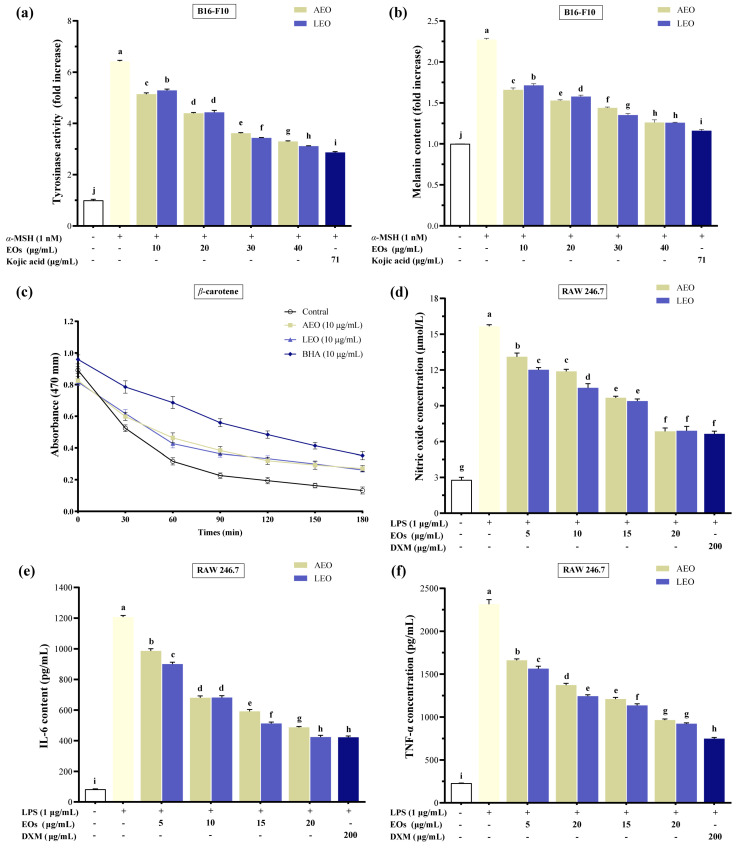
Effects of AEO and LEO on (**a**) cellular tyrosinase activity and (**b**) melanin content in *α*-MSH-stimulated B16 cells. (**c**) The antioxidant efficacy of AEO and LEO was evaluated utilizing a *β*-carotene/linoleic acid bleaching assay. Effects of AEO and LEO on (**d**) NO synthesis, (**e**) IL-6 levels, and (**f**) TNF-*α* levels in LPS-activated RAW 264.7 macrophage cells. The results are expressed as mean and standard deviation (*n* = 3). Statistical data discrepancies were confirmed at *p* < 0.05 and denoted by distinct lowercase letters in the graph.

The influence of two EOs, AEO and LEO, on cellular tyrosinase activity and melanin production was explored in this study. B16/F10 melanoma cells were exposed to *α*-MSH (1 nM) for 48 h and concurrently treated with EOs at 10, 20, 30, and 40 µg/mL doses. The results in Figure 2a illustrate a six-fold enhancement in cellular tyrosinase activity in α-MSH-treated cells relative to the control group. AEO and LEO exhibited a dose-dependent suppression of α-MSH-induced tyrosinase activity, with the most pronounced inhibitory effects noted at the 40 µg/mL concentration. The inhibitory effect of LEO on tyrosinase was significantly higher (*p* < 0.05) than that of AEO at the highest inhibitory concentration. In particular, both AEO and LEO demonstrated significant tyrosinase inhibitory activity, with 87.89 ± 0.61% and 93.11 ± 0.45%, respectively, relative to the positive control, kojic acid at 71 µg/mL, on the tyrosinase inhibition rate.

The melanin levels were about 2.2 times higher in the *α*-MSH-incubated B16/F10 cells than in the controls (Figure 2b). AEO and LEO effectively reduced *α*-MSH-induced melanin production depending on the application dosage. At a 40 µg/mL concentration, both AEO and LEO significantly (*p* < 0.05) inhibited the melanin synthesis rate by 55.33 ± 0.33% and 55.47 ± 1.41%, respectively. There was no significant difference in melanin content inhibition at the highest inhibitory concentration between AEO and LEO. The anti-melanogenic activities of AEO and LEO, relative to a 71 µg/mL kojic acid inhibition rate, were 91.30 ± 0.68% and 91.03 ± 2.87%, respectively.

#### 2.2.2. In Vitro Antioxidant Activity

The antioxidant properties of AEO and LEO were evaluated using the *β*-carotene–linoleic acid model and compared with those of the positive control antioxidant, BHA (Figure 2c). The antioxidant activity was measured as the percentage of oxidized *β*-carotene. At a dose of 10 µg/mL, both AEO and LEO effectively retarded the decrease in 470 nm absorbance over 180 min and inhibited the oxidation of linoleic acid compared to the control. The antioxidant capacities of oxidized *β*-carotene (%) for the control, AEO, LEO, and BHA were 85.23 ± 1.03%, 67.25 ± 1.09%, 68.02 ± 0.71%, and 63.27 ± 0.96%, respectively. AEO and LEO exhibited slightly lower antioxidant capacities than BHA in preventing linoleic acid oxidation. No significant variation in antioxidant capabilities was detected between AEO and LEO.

#### 2.2.3. In Vitro Anti-Inflammatory Activity

The LPS-induced RAW264.7 cell inflammation model was employed to assess the anti-inflammatory properties of AEO and LEO. The cytotoxic effects of AEO and LEO on RAW 264.7 cells were assessed using the standard CCK-8 assay, in which the cells were subjected to varying doses of EOs (ranging from 5 to 30 µg/mL) to identify a non-toxic concentration range (Figure S3). Cell viability was unaffected at the maximum tested dose of 20 µg/mL for both AEO and LEO. Accordingly, RAW 264.7 cells were subjected to LPS (1 µg/mL) treatment for 24 h in conjunction with AEO and LEO at doses of 5, 10, 15, and 20 µg/mL for further experimentation.

The NO levels were indirectly measured by examining the impact on nitrite production in an LPS-activated RAW 264.7 macrophage culture, with the results depicted in Figure 2d. AEO and LEO demonstrated a dose-dependent reduction in NO production. AEO and LEO inhibited NO production by 56.24 ± 2.16% and 56.02 ± 2.63%, respectively, at a 20 µg/mL concentration. The specific inhibition of DXM (200 µg/mL) was used as a positive control, inhibiting 57.58 ± 1.69% of NO production. Notably, the suppressive effects of AEO and LEO at a dose of 20 µg/mL on NO levels were not significantly different from those of DXM.

The supernatant from cells subjected to different concentrations of AEO and LEO was utilized to quantify pro-inflammatory cytokines (IL-6 and TNF-*α*). As depicted in Figure 2e,f, AEO and LEO significantly down-regulated the LPS-induced cellular TNF-α and IL-6 levels relative to the control group (*p* < 0.05). At a dose of 20 µg/mL, AEO and LEO exhibited inhibition rates of 59.65 ± 1.11% and 64.87 ± 0.57% for IL-6, and 58.29 ± 0.69% and 60.10 ± 0.51% for TNF-*α,* respectively. The inhibitory capability of LEO on IL-6 was significantly higher (*p* < 0.05) than that of AEO. LEO exhibited a suppressive impact on IL-6 comparable to that of DXM, with relative inhibition rates of 99.86 ± 1.69%. Additionally, there was no significant difference in the TNF-*α* inhibition rates between AEO and LEO. The TNF-*α* inhibition rates of AEO and LEO, relative to DEX, were 86.21 ± 1.02% and 88.88 ± 0.75%, respectively.

### 2.3. Network Pharmacology Prediction

#### 2.3.1. Putative Targets of EOs for the Treatment of Skin Pigmentation and Inflammation

A total of 622 targets of AEO and 685 targets of LEO were identified from the SuperPred, SwissTargetPrediction, and TargetNet databases after eliminating duplicate entries. Disease-related targets were acquired by consulting the OMIM, DisGenet, and GeneCards databases. A total of 1382 target genes were identified for skin pigmentation and 1870 target genes were identified for skin inflammation. Upon comparing the skin pigmentation-related targets with the active-compound-related targets in AEO and LEO, 140 and 157 intersecting targets were identified, respectively (Figure S5a). Similarly, 235 and 255 intersecting targets have been found between the active-compound-related targets in AEO and LEO and the skin inflammation-related targets (Figure S5b). Detailed information on the intersecting targets between compound-related targets and disease-related targets is listed in Table S1.

#### 2.3.2. Compound–Target Interaction Network

**Figure 3:**
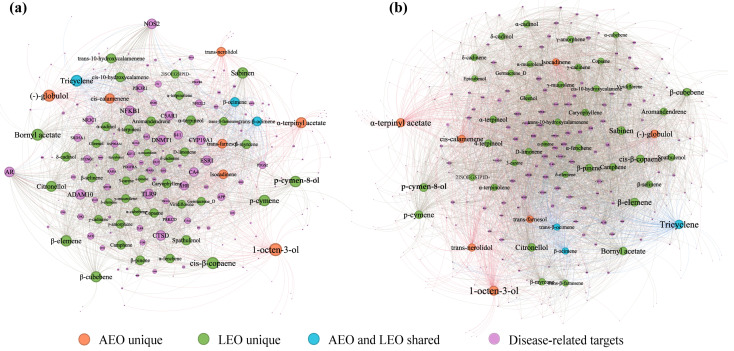
Compound–target interaction network of AEO and LEO with (**a**) skin pigmentation and (**b**) skin inflammation. Node size is relative to degree.

To further understand the interaction mechanisms between active compounds and disease targets, compound–target interaction networks were constructed to connect the active compounds with the screened intersection protein targets (Figure 3a,b). The EO–skin pigmentation network contained 208 nodes and 2457 edges, with a network heterogeneity score of 0.864 (Figure 3a). The EO–skin inflammation network had 308 nodes and 4945 edges, with a network heterogeneity score of 1.069 (Figure 3b). A significant association existed between the active constituents of AEO and LEO and targets related to diseases. The degrees (*k*) of the EO–skin pigmentation and inflammation interaction networks were 1.50 and 1.61, respectively, with a *p*-value below 0.01 (Figure S6a,b). A strong correlation between the betweenness centrality and degree indicates a quadratic fit (Figure S6c,d). The networks demonstrate a nearly scale-free property and a power-law degree distribution, showing that key nodes are critical in the compound–target interaction networks. The topology analysis results were calculated to explore the main compounds that signify a closer correlation with disease-related protein targets (Table S2).

The top five core compounds were further screened based on the MCC value. Core nodes of AEO in the EO–skin pigmentation network were identified as cis-*β*-copaene, bornyl acetate, tricyclene, p-cymene, and p-cymen-8-ol. The core compounds of LEO in the EO–skin pigmentation network were 1-octen-3-ol, cis-*β*-copaene, (−)-globulol, bornyl acetate, and p-cymen-8-ol. The core compounds of AEO in the EO–skin inflammation network were tricyclene, *β*-element, p-cymen-8-ol, bornyl acetate, and p-cymene. The core compounds for LEO in the EO–skin inflammation network were confirmed as 1-octen-3-ol, *α*-terpinyl acetate, *β*-elemene, p-cymen-8-ol, and (−)-globulol.

#### 2.3.3. PPI Network Construction

**Figure 4:**
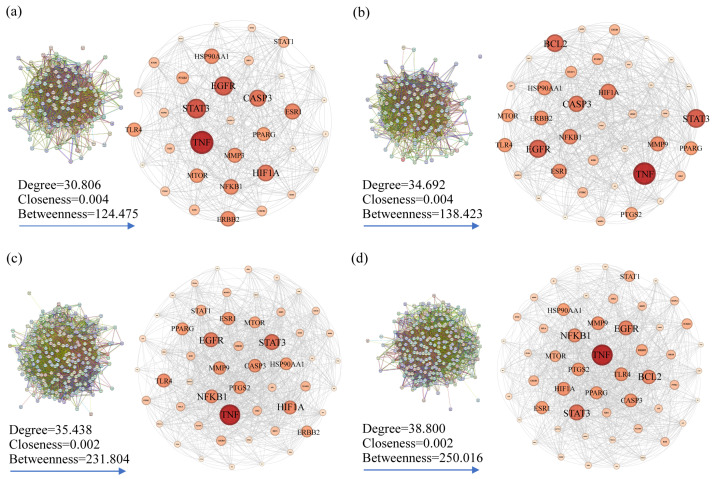
Protein–protein interaction (PPI) network constructed by using STRING database. (**a**) Skin pigmentation-related PPI network of AEO. (**b**) Skin pigmentation-related PPI network of LEO. (**c**) Skin inflammation-related PPI network of AEO. (**d**) Skin inflammation-related PPI network of LEO. Nodes signify proteins, with colors ranging from pink to red indicating the extent of binding between them. Edge signifies protein–protein interaction.

The PPI network was acquired via the STRING online database to illustrate the relationships between the intersection targets, and the core targets were screened according to the median values of the degree, BC, and CC. For skin pigmentation, the AEO PPI network had 35 nodes connected by 479 edges (Figure 4a), and the top five core targets were identified as TNF, STAT3, EGFR, CASP3, and HIF1A. The LEO PPI network comprises 39 nodes linked by 575 edges (Figure 4b), and the top five core targets were identified as TNF, STAT3, EGFR, BCL2, and CASP3. The core targets of AEO and LEO for addressing skin pigmentation concerns exhibit a high degree of similarity, with 33 shared targets constituting 80.5% of the total core target set.

Regarding skin inflammation, the AEO PPI network encompassed 53 nodes and 887 edges (Figure 4c), and the top five core targets were TNF, STAT3, EGFR, HIF1A, and NFKB1. The LEO PPI network consists of 53 nodes connected by 598 edges (Figure 4d), and the top five core targets were TNF, STAT3, EGFR, BCL2, and NFKB1. The core targets identified in AEO and LEO for addressing skin inflammation also display a substantial overlap, with 49 shared targets representing 86.5% of the total core target set. The results indicated that AEO and LEO have similar pharmacological effects in alleviating skin pigmentation and inflammation.

#### 2.3.4. Bioinformatic Enrichment

Core targets were analyzed using the DAVID online database for GO and KEGG pathway enrichment to elucidate their mechanisms. Detailed results are presented in Tables S3–S7. In the skin pigmentation treatment, AEO and LEO identified 299 and 203 items, respectively, including 119 and 127 for BP, 39 and 49 for MF, 19 and 27 for CC, and 122 and 128 for signaling pathways. For skin inflammation treatment, AEO and LEO identified 257 and 258 items, respectively, encompassing 179 and 182 in biological processes, 49 and 51 in molecular functions, 26 and 25 in cellular components, and 130 and 128 in signaling pathways. The most significant GO terms and top 10 and 30 KEGG signaling pathways, ranked by adjusted *p*-value, are presented in Figure S7a–d.

**Figure 5:**
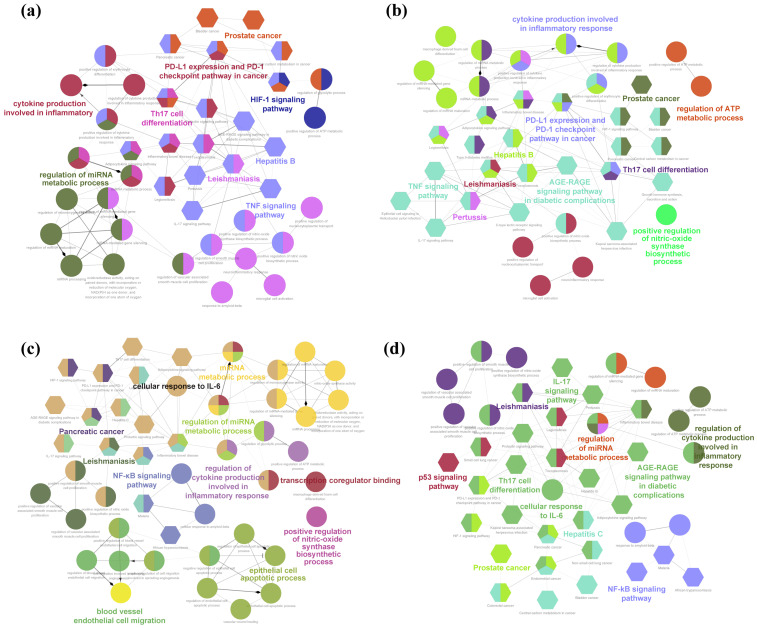
The interaction network of GO terms and KEGG pathways generated using ClueGo plugin in Cytoscape. (**a**) Skin pigmentation-related interaction network of AEO. (**b**) Skin pigmentation-related interaction network of LEO. (**c**) Skin inflammation-related interaction network of AEO. (**d**) Skin inflammation-related interaction network of LEO. Similar signaling terms are represented as a cluster by nodes of the same color. A node is involved in more than one cluster of terms if it contains different colors.

The ClueGO plugin was employed to cluster analogous terms and functions of the core targets, thereby uncovering representative pharmacological effects. For AEO–skin pigmentation (Figure 5a), the potential biological effects identified by GO were mainly associated with the regulation of miRNA metabolic processes and cytokine production in inflammatory responses. Moreover, the highly enriched signaling pathways primarily included the PD-L1 expression and PD-1 checkpoint pathway in cancer, Th17 cell differentiation, the TNF signaling pathway, and the HIF-1 signaling pathway. For LEO–skin pigmentation (Figure 5b), the potential biological effects included the regulation of ATP metabolic processes, cytokine production involved in inflammatory responses, and the positive regulation of NO synthase biosynthetic process. The signaling pathways mainly consisted of Th17 cell differentiation, the TNF signaling pathway, PD-L1 expression and PD-1 checkpoint pathway in cancer, leishmaniasis, Hepatitis B, prostate cancer, the AGE-RAGE signaling pathway in diabetic complications, and pertussis.

In AEO–skin inflammation (Figure 5c), the potential biological effects were observed, including blood vessel endothelial cell migration, the epithelial cell apoptotic process, the regulation of cytokine production involved in inflammatory response, cellular response to IL-6, and the regulation of miRNA metabolic process. The predominant signaling pathways included those related to pancreatic cancer, prostate cancer, leishmaniasis, and the NF-κB signaling pathway. In LEO–skin inflammation (Figure 5d), the observed biological effects included the regulation of miRNA metabolic process, the regulation of cytokine production involved in inflammatory response, and cellular response to IL-6. The primary signaling pathways encompassed the p53 signaling pathway, IL-17 signaling pathway, Th17 cell differentiation, the AGE-RAGE signaling pathway in diabetic complications, the NF-κB signaling pathway, prostate cancer, and Hepatitis C.

#### 2.3.5. Molecular Docking Analysis

Based on the results of the network pharmacology topology analysis, the core active compounds in AEO (cis-*β*-copaene, p-cymen-8-ol, bornyl acetate, p-cymene, and tricyclene) and LEO (1-octen-3-ol, cis-*β*-copaene, (−)-globulol, p-cymen-8-ol, and bornyl acetate) were screened. Molecular docking was performed with these active compounds and the core proteins associated with skin pigmentation and inflammation, including TNF (PDB ID: 7KPA), STAT3 (5AX3), EGFR (5UGC), CASP3 (5IBP), HIF1A (4H6J), BCL2 (6GL8), and NFKB1 (7RG5). Since D-limonene and *α*-pinene constitute the most abundant ingredients of AEO and LEO, they were also selected for molecular docking. The interactions between the active compounds and the disease-related proteins are shown in Table 2.

**Figure 6:**
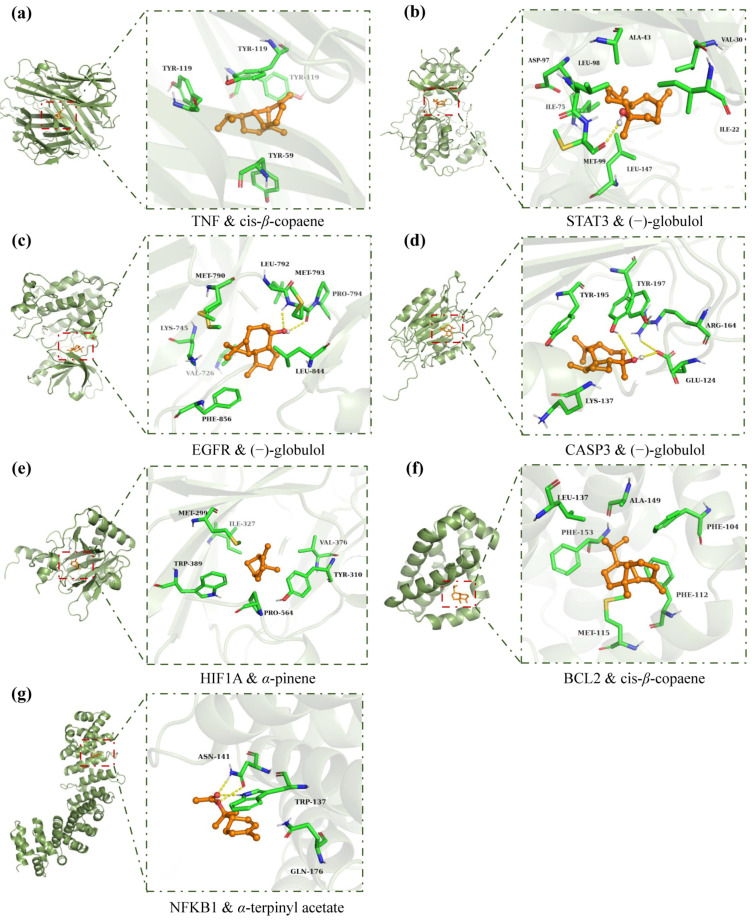
Docking complexes of the main targets and their most strongly bound compositions. (**a**) TNF and cis-*β*-copaene, (**b**) STAT3 and (−)-globulol, (**c**) EGFR and (−)-globulol, (**d**) CASP3 and (−)-globulol, (**e**) HIF1A and *α*-pinene, (**f**) BCL2 and cis-*β*-copaene, and (**g**) NFKB1 and *α*-terpinyl acetate.

The findings show that the binding energies of component–target docking were all below −5 kcal/mol, implying that the active compounds in AEO and LEO could stably bind to the disease-related proteins. Among them, the binding energies of some active compounds to proteins were below −7.25 kcal/mol, indicating a strong binding affinity. For clarity, Figure 6 illustrates the optimal docking of receptors and ligands. The results suggest that these ingredients and proteins are the primary active components and targets for treating skin pigmentation and inflammation in AEO and LEO.

## 3. Discussion

### 3.1. EO Yield and Composition between Torreya Aril and Leaves

Plant EOs are volatile mixtures with a low molecular weight that are biosynthesized in different plant organs. Based on the number of carbon atoms, terpenes primarily consist of hemiterpenes, monoterpenes, sesquiterpenes, diterpenes, triterpenes, and tetraterpenes. Notably, monoterpenes, sesquiterpenes, and their oxygenated derivatives are the predominant chemical constituents of EOs [18]. In this study, AEO was found to contain a significant amount of monoterpene hydrocarbons, with the most substantial components identified as *α*-pinene (39.15%), D-limonene (25.57%), 3-carene (3.89%), and *δ*-cadinene (3.42%). These results indicate variations in the relative abundance of the primary components relative to those documented in the article. Wang et al. (2022) reported that D-limonene (27.01%), *α*-pinene (7.66%), 3-carene (4.62%), and trans-*β*-ocimene (4.16%) were the four most abundant components of AEO [6]. According to the report by Niu et al. (2010), EOs from arils exhibited high contents of *α*-pinene, D-limonene, limonene glycol, and (*E*)-carveol, amounting to 34.20%, 27.58%, 3.64%, and 2.50%, respectively [4]. The identified variations in the chemical composition and relative abundance of AEO can be ascribed to many sources. These factors encompass genetic potential, germplasm characteristics, growth conditions, developmental stages, organic differentiation, and extraction methods, all of which could significantly influence the composition of EOs [19].

The type of plant organ is considered one of the most critical factors influencing the quality and quantity of EOs in EO-bearing plants [20]. Llorens-Molina et al. (2015) examined the variation in EO components across different organs of Spanish *Artemisia absinthium* populations [21]. The findings indicated that the aerial parts predominantly contained oxygenated monoterpenes (81.4–89.1%), with the main constituents being (*Z*)-epoxyocimene, (*Z*)-chrysanthemyl acetate, and linalool, whereas root EOs had higher proportions of monoterpenes (43.8–55.1%) with the primary components being *β*-myrcene and *α*-terpinene. In this study, although many compounds were identical between the EOs from the Torreya leaves and arils, the yield and concentrations of these compounds differed significantly. One possible explanation is that the leaves and arils possess different EO-secreting glands. Plant volatiles are produced via distinct biochemical pathways within specialized secretory structures, including secretory glands, cavities, channels, and glandular trichomes. Consequently, plant EOs exhibit significant variations in both quantity and quality across different organs. Dodos et al. (2021) documented the chemical composition of EOs and the micromorphological traits of different organs from three species of *Satureja* spp. [20]. The findings revealed significant differences among plant organs regarding the quantity and types of glandular trichomes, indicating that the heterogeneity in EO composition may be linked to the density and profile of glandular trichomes across different plants and their respective organs. Again, volatile variability is particularly evident in entomophilous flowers, in which these compounds serve as orientation cues for pollinators. Consequently, the volatiles emitted by these flowers differ significantly from those in other plant parts. Generally, terpenoid levels are higher in reproductive structures, which directly influence the attraction of pollinators to insects [22,23]. The higher monoterpene content in the EO of the aril and the increased sesquiterpenoid content in the EO of leaves may reflect the organ-specific functional strategies of *T. grandis*, which optimize the metabolic components crucial for their defensive or attractive roles. Similar patterns were observed in various growth stages of *Lavandula pinnata*, whose flowers contain higher monoterpenoid levels than its leaves and stems [22].

It is important to note that although the yield of LEO is one-fifth that of AEO, extraction from leaves offers distinct advantages. *T. grandis* is an evergreen species whose leaf-derived EO remains consistent regardless of seasonal changes. Furthermore, regular pruning helps maintain the Torryea’s optimal structure. It produces significant waste, such as branches and leaves, which can be used as additional raw materials for EO extraction. Consequently, utilizing Torreya leaves as an auxiliary source for EO production could overcome the seasonal constraints of aril-derived EO production and effectively use pruning waste. However, further analysis is required to determine if LEO can be a viable alternative to AEO based on their bioactivity differences.

### 3.2. Biological Activities of EOs from Torreya Leaves and Aril

EOs are extensively utilized in contemporary skincare products due to their complex active compounds, potent fragrance characteristics, and natural origins. Along with providing delightful aromas in various products, they can serve as preservatives and active agents [24,25]. Moreover, the volatile compounds in EOs are lipophilic and have low molecular weights, enabling them to penetrate cell membranes rapidly. These volatile compounds contribute to numerous skincare benefits, including but not limited to anti-melanogenic, antioxidant, and anti-inflammatory properties, making them invaluable in cosmetics [26].

Melanin is synthesized by melanocytes located in the basal layer of the epidermis and is crucial in determining skin color. Excessive melanin production can lead to dermatological conditions, including melasma, freckles, age spots, etc., and may be associated with skin cancer [27]. Tyrosinase is the rate-limiting enzyme in melanin production, and the most effective method to impede skin pigmentation is by inhibiting tyrosinase [28]. Therefore, discovering tyrosinase inhibitors is essential for skin whitening and depigmentation products in the pharmaceutical and cosmetics industries. Feng et al. (2019) reported the inhibiting effects of AEO on mushroom-derived tyrosinase, demonstrating that AEO exhibited superior tyrosinase inhibition compared to lemon EO [7]. In this study, the comparative inhibitory effects of AEO and LEO on tyrosinase and melanin production in *α*-MSH-stimulated B16 cells were investigated, and the results indicated that AEO and LEO act as tyrosinase inhibitors and reduced melanin production. Functional hypopigmentation agents can reduce melanin synthesis in melanocytes by inhibiting cellular tyrosinase activity [11]. Several EOs with similar main active components to AEO and LEO, such as *Alpinia nantoensis* EO, *Juniperus phoenicea* EO, *Pistacia lentiscus* EO, and *Citrus maxima* EO, whose main active components are *α*-pinene and/or D-limonene, have been reported to possess anti-tyrosinase and anti-melanogenic activities [29,30,31,32]. *α*-pinene and D-limonene have been confirmed to have tyrosinase inhibitory potential, with IC_50_ values of 97.45 µg/mL and 74.24 µg/mL, respectively [31]. The inhibitory activity of tyrosinase in Torreya EOs may be ascribed to the structure–activity relationships of their ingredients. Specific terpenoids, such as α-pinene, α-terpineol, and 1,8-cineole, feature phenolic structures in which a hydroxyl group is attached to the para position of the phenolic ring. This hydroxyl group acts as an electron donor during the reaction, a critical factor in their anti-tyrosinase activity [11]. In this study, at a 40 µg/mL concentration, AEO and LEO exhibited inhibition rates of 48.56 ± 0.48% and 51.35 ± 0.51% on intracellular tyrosinase, respectively. These inhibition rates are superior to those of *α*-pinene and D-limonene, which were discussed earlier. Previous research has indicated that complex mixtures of compounds often perform better than single, isolated compounds [17]. This observation aligns with findings in traditional Chinese medicine, according to which plant extracts show reduced efficacy when fractionating and purifying [33,34]. Antioxidants exhibit anti-melanogenic capabilities owing to their capacity to scavenge free radicals and interact with melanogenic intermediates [13]. Several antioxidants, including ascorbic acid derivatives and reduced glutathione (GSH), are utilized as melanogenesis inhibitors [11]. Numerous studies have indicated that plant EOs are ideal natural sources of antioxidants, with many aromatic components contributing to their antioxidant activity [35,36]. A previous study evaluated approximately a hundred pure components of EOs to assess their antioxidant efficacy. This research showed that monoterpene hydrocarbons (such as terpinolene and *α*- and *γ*-terpinene) and oxygenated monoterpenes (including phenols and allylic alcohols) exhibited appreciable antioxidant activity. In contrast, sesquiterpene hydrocarbons and non-isoprenoid components demonstrated relatively low levels of antioxidant activity [37]. In this study, both AEO and LEO demonstrated excellent potential for inhibiting lipid peroxidation. AEO and LEO contain high levels of monoterpene hydrocarbons (78.43 and 48.84%, respectively), suggesting that these constituents may be the principal contributors to the antioxidant action of EOs. Additionally, *α*-pinene and D-limonene, the principal compounds of AEO and LEO, have been identified to possess significant antioxidant abilities [38,39]. Feng et al. (2019) assessed the antioxidant capabilities of AEO by a 2,2-diphenyl-1-picrylhydrazyl (DPPH) radical scavenging assay, revealing an IC_50_ value of 0.88 µg/mL, which signifies superior antioxidant activity compared to that of vitamin C and lemon EO [7]. Yu et al. (2016) evaluated the antioxidant activity of AEO by three distinct methodologies: a DPPH free radical scavenging assay, a β-carotene/linoleic acid bleaching method, and a thiobarbituric acid reactive species assay, thereby substantiating the antioxidant properties of AEO [5]. There was no significant difference in lipid antioxidant activity between LEO and AEO, suggesting that *T. grandis* leaves are also a valuable biological resource for EOs possessing significant antioxidant activity.

Inflammation is considered a primary catalyst for pigmentary diseases. In the post-inflammatory skin, several interleukins and other inflammatory mediators either promote or inhibit the proliferation and differentiation of human epidermal melanocytes, directly or indirectly affecting melanogenesis-related gene expression and thereby regulating skin pigmentation [14]. The inhibition of acute or chronic skin inflammation may be one of the ways to prevent skin melanin deposition. The overproduction of pro-inflammatory mediators such as prostaglandin E2 (PGE2) and NO, along with cytokines including TNF-*α*, cyclooxygenase 2 (COX-2), IL-6, and interleukin 1*β* (IL-1*β*), serves as a crucial indicator for identifying early inflammatory reactions [14,40]. Inhibiting the release of these molecules has become a crucial approach to treating inflammation. NO is a crucial cellular signaling molecule involved in various pathological and physiological processes, including both acute and chronic inflammation. The overexpression of NO may stimulate the synthesis of pro-inflammatory cytokines. Moreover, NO is essential in melanogenesis and represents a potential target for inhibiting UV-induced melanogenesis [13]. This study demonstrated that LEO and AEO, in addition to reducing NO production, also suppressed the production of the pro-inflammatory cytokines IL-6 and TNF-*α*. Many EOs have demonstrated a correlation between anti-inflammatory qualities and their capacity to inhibit pro-inflammatory cytokines. The predominant components of EOs, such as monoterpenes and sesquiterpenes, effectively decrease inflammatory responses and regulate inflammatory cytokines due to their distinctive absorption and quick response [41,42]. In a study on the in vitro anti-inflammatory activity of twenty-one citrus peel EOs, the seven single compounds, i.e., *α*-pinene, D-limonene, myrcene, *β*-ocimene, linalool, linalool oxide, and *α*-terpineol, which were similar to the main component of AEO and LEO, were identified as contributors to anti-inflammatory activity [43]. For instance, *α*-pinene was reported to suppress the protein expression of inflammatory mediators, including nuclear factor-kappa B (NF-κB), TNF-*α*, and IL-6, in human skin epidermal keratinocytes (HaCaT) and reduced the production of ROS [39]. Moreover, D-limonene has been documented to modulate the activities of inflammatory cytokines, including TNF-*α*, IL-1*β*, and IL-6 [38]. The anti-inflammatory properties of AEO and LEO may be ascribed to their active constituents. Overall, AEO and LEO demonstrate similar inhibitory effects on in vitro antioxidant activities, melanogenesis inhibition, and anti-inflammatory activities. From a bioactivity perspective, Torreya leaves could be an alternative material source for producing Torreya aril EO.

### 3.3. Molecular Mechanisms of Torreya EO Treatment for Skin Pigmentation and Inflammation

The biological activities of EOs are commonly assessed using pharmacological experimental models, with the activity often attributed to the most abundant/meaningful compounds in the composition. However, this approach overlooks the potential contributions of the less prevalent constituents of EOs to their biological activity and the possible biological interactions among various components, thus disregarding the multi-component and multi-target attributes of EOs. Network pharmacology is a contemporary analytical method that integrates high-throughput omics data, computational simulations, and network databases [17]. This approach enables a comprehensive examination of EOs, considering all possible active constituents and clarifying the molecular mechanisms and pathways associated with the targets.

In this study, numerous potential targets associated with skin pigmentation and inflammation within the compound–target network exhibited tight binding patterns with the compounds in AEO and LEO, suggesting their potential efficacy in treating these conditions and exhibiting multi-component and multi-target characteristics. Based on the topology analysis of the compound–target network, the proportions of the most significant compounds for addressing skin pigmentation and inflammation from AEO and LEO range from 0.10% to 1.27%. In contrast to mainstream ingredients such as *α*-pinene and D-limonene, which collectively account for over 50% of the composition, the minor compounds in EOs demonstrate a greater affinity for disease-related targets. Similar outcomes have been observed in previous research. For example, Wang et al. (2023) analyzed the potential bioactivities of EOs derived from *Artemisia argyi* and *Artemisia verlotorum*. While compounds such as neointermedeol (with a relative content ranging from 2.79% to 9.39%), caryophyllene (1.02% to 8.73%), and caryophyllene oxide (3.42% to 23.7%) were identified as predominant components of the essential oils, a network pharmacology analysis showed that less abundant compounds like methylaugenol (0.17% to 0.21%), 1-octen-3-ol (0.2% to 0.34%), *o*-cymene (0.17%), chamazulene (0.53% to 1.25%), and eugenol (0.45% to 0.84%) were the core active constituents [44].

The PPI networks and bioinformatic enrichment results indicated that treating skin pigmentation and inflammation with AEO and LEO involves multiple targets, biological processes, and signaling pathways. Interestingly, there was a significant overlap in the core targets, processes, and pathways related to skin pigmentation and inflammation. This suggests that shared molecular mechanisms potentially exist between the two EOs. The GO enrichment results related to skin pigmentation prominently featured cytokine production within inflammatory responses. Several inflammatory mediators have been shown to influence melanogenesis-related signaling pathways by binding to specific receptors, which either promote or inhibit the expression of genes connected with melanogenesis and regulating skin pigmentation processes. Fu et al. (2020) reported that inflammatory cytokines such as interleukin-18 (IL-18), interleukin-33 (IL-33), granulocyte-macrophage colony-stimulating factor (GM-CSF), interferon-γ (IFN-γ), and PGE2 have been shown to stimulate melanogenesis [14]. Signaling pathways, including Th17 cell differentiation and the TNF signaling pathway, were also observed to be linked to skin pigmentation. Th17 cells, a recently discovered subset of T cells, secrete various cytokines such as IL-6, interleukin-17 (IL-17), interleukin-21 (IL-21), and interleukin-22 (IL-22), which could contribute to inflammation by secreting IL-17, IL-6, and TNF-*α* [14,45]. 

In addition to the targets of TNF, STAT3 and EGFR are among the top three core targets within the four PPI networks. The STAT protein family comprises transcription factors crucial for cytokine and growth factor receptor signaling, significantly influencing cell growth, survival, differentiation, and motility [46]. STAT3, a prevalent member of the STAT protein family, can be activated by *α*-MSH in melanocytes, leading to phosphorylation and significantly suppressing the transcription of tyrosinase genes, thereby inhibiting melanin production [47]. In addition to this direct mechanism, the activation of STAT3 within the miR-7/STAT3 pathway can indirectly trigger the paracrine axis, influencing melanin secretion [48]. EGFR is critical in skin biology and inflammatory responses [49]. Chronic skin inflammation has been associated with the up-regulation of EGFR and its ligand expression [49,50]. EGFR signaling also inhibits melanogenesis; for instance, up-regulated EGFR and Akt phosphorylation levels can reduce the increase in cellular melanogenesis induced by UV radiation [51]. Besides this direct mechanism, epidermal growth factor (EGF) stimulates cell growth by binding to EGFR, promoting skin lightening and preventing post-inflammatory pigmentation through accelerated wound healing [52]. We also observed that results from inflammation-related signaling pathways were involved in the NF-κB signaling pathway. NF-κB is a pivotal mediator of inflammatory responses, composed of distinct subunits encoded by five independent genes: NF-κB1 (also known as p50), NF-κB2 (p52), RelA (p65), RelB, and c-Rel. NF-κB can induce the expression of various pro-inflammatory genes and participate in inflammasome regulation [53]. In addition, NF-κB is crucial in indirectly regulating melanin synthesis, potentially involving autophagy to reduce melanin synthesis in melanocytes [54].

According to the molecular docking results, the binding energies of all core targets and core compounds were between −5.0 and −9.6 kcal/mol, indicating a good performance and a strong binding affinity. Binding energy values below −4.25 kcal/mol, −5.0 kcal/mol, and −7.0 kcal/mol are widely recognized as indicative of certain, good, and strong binding interactions between the ligand and the receptor, respectively [55]. The active compound (−)-globulol demonstrates a strong binding affinity, exhibiting the lowest binding energies with STAT3, CASP3, and EGFR. It forms hydrogen bonds with their respective amino acid residues. *α*-terpinyl acetate exhibits the lowest binding energies with NFKB1, forming hydrogen bonds with amino acid residues of ASN-141 and TRP-137. Previous research has indicated that oxygenated monoterpenes, which feature oxygen functional groups (-OH, -CHO, and -C=O) in their molecular structures, can act as electron donors or acceptors, forming intermolecular hydrogen bonds or other chemical reactions, typically exhibiting enhanced biological activities [56]. Unlike (−)-globulol and *α*-terpinyl acetate, cis-*β*-copaene and *α*-pinene feature a fully carbon-based ring structure and closely interact with the hydrophobic binding cavities on protein targets. Active ingredients, such as (−)-globulol [57,58], *α*-terpinyl acetate [59], bornyl acetate [60,61], and p-cymene [62], identified through molecular docking have been confirmed to exhibit anti-inflammatory or tyrosinase inhibitory activities. Besides D-limonene and *α*-pinene, these low-content compounds significantly address skin pigmentation and inflammation in AEO and LEO. Notably, there are some limitations to this result. For instance, alongside the intrinsic false positive rate associated with molecular docking, contemporary network information technology necessitates additional improvements, and the precision of the database information requires scientific validation.

## 4. Materials and Methods

### 4.1. Plant Material and EO Hydrodistillation

Plant material, including fresh arils and leaves of *T. grandis*, were collected in October 2023 from the Torreya plantation of Qiaoming Gongfei Co., Ltd. located in Huangshan District, Anhui, Eastern China (30°13′17″ N, 118°7′33″ W). The aril was carefully cleaned, crushed using a metal blender, and subjected to hydrodistillation via a Clevenger-type device. The hydrodistillation method entailed the extraction of 100 g of aril and 200 g of leaves under specified conditions: a 1:8 material:liquid ratio in a 2 L round-bottom flask, 1 kW of extraction power using a heating jacket, and 2 h of extraction time (starting from condensation). Subsequently, the acquired AEO and LEO were recovered, dehydrated with anhydrous Na_2_SO_4_, and preserved in amber vials at 4 °C until further analysis. The yield of EO (mg/g) was determined as the mass percentage of EO (mg) concerning the weight of the raw sample (g).

### 4.2. Chemical Analysis

The composition of AEO and LEO was evaluated using GC-MS (TRACE1310 and TSQ8000; Thermo Scientific, Waltham, MA, USA) equipped with a TG-5SILMS (Thermo Scientific) capillary column. Before analysis, the EO samples were dissolved in *n*-hexane (1:100, *v*/*v*), and 1 µL of the resultant solution was injected into the GC-MS apparatus. The GC temperature program involved an isothermal hold at 40 °C for 1 min, followed by a ramp from 40 to 120 °C at a rate of 4 °C/min, and a final hold at 250 °C at a rate of 5 °C/min. The front injection temperature was set at 240 °C, while the ion source temperature was 260 °C. Each component was identified by its mass spectral fragmentation patterns, referencing the NIST17 mass spectral library. The retention indices (RIs) were computed and compared to a homologous sequence of *n*-alkanes (C_8_–C_40_) to confirm the identification. The raw data of the metabolite have been uploaded to the MetaboLights database (Accession Number: MTBLS10119).

### 4.3. Determination of Antioxidant Activity with β-Carotene Bleaching Assay

The antioxidant effectiveness of AEO and LEO was assessed using the *β*-carotene bleaching method described by Chou et al. (2018) [13]. In this process, 0.1 mg of *β*-carotene was initially dissolved in 5 mL of chloroform; then, 1 mL of this solution was combined with 20 µL of linoleic acid and 200 mg of Tween 80. The resulting solution was evaporated under a nitrogen stream and subsequently reconstituted in 50 mL of oxygenated water. For the experiment, 40 mL of the samples (10 µg/mL) were mixed with 960 mL of the *β*-carotene–linoleic acid emulsion. The blank control consisted of an equivalent volume of methanol in place of the test samples, while butylated hydroxyanisole (BHA) at a concentration of 10 µg/mL functioned as the positive control. The reaction mixture was incubated at 50 °C, and the absorbance was recorded at 470 nm. The absorbance readings of all samples were initially recorded and thereafter at 30 min intervals for a total duration of 180 min.

### 4.4. Cell Line Culture and Cell Viability Assay

The B16/F10 melanoma cell line and RAW 264.7 macrophages were obtained from LinMei Biotechnology Co., Ltd. (Hefei, China). These cell lines were cultured in DMEM supplemented with 10% FBS, 2 nM L-glutamine, 100 mg/mL streptomycin, and 100 U/mL penicillin. The cells were maintained in a controlled environment at a temperature of 37 °C with a humidified atmosphere containing 5% CO_2_. Subculturing was performed every 3 to 4 days to ensure continuous exponential growth.

B16/F10 melanoma cells were introduced into each well of a 96-well plate at a density of 5 × 10^3^ cells per well for the purpose of conducting a cell viability assay. Following an incubation period of 24 h, different concentrations (10, 20, 30, 40, and 50 µg/mL) of both Torreya EOs and *α*-MSH (1 nM) were placed in each individual well. The plate was then subjected to further incubation for a duration of 72 h. Similarly, RAW264.7 cells were seeded in separate wells of another set of 96-well plates at a density level equivalent to that of approximately 3 × 10^5^ cells density per well. These cells were subsequently treated with varying concentrations (5, 10, 15, 20, and 30 µg/mL) of Torreya EOs as well as lipopolysaccharides (LPSs), specifically at a concentration level equaling 1 µg/mL. The aforementioned plates containing the treated RAW264.7 cells were maintained under controlled conditions, with a temperature setting at 37 °C within a humidified atmosphere comprising 95% air and 5% CO_2_ for 20 h. The CCK-8 kit (Sigma, St. Louis, MI, USA) was employed to evaluate the cell viability levels exhibited by both the B16/F10 melanoma cells and the RAW264.7 cells. Subsequently, the absorbance values were recorded at 450 nm.

### 4.5. Cellular Melanin Content and Tyrosinase Activity Assay

The quantification of melanin content in B16/F10 melanoma cells was conducted following the methodology described by Chou et al. (2018) [13], with minor adjustments. B16/F10 cells were exposed to varying concentrations (10, 20, 30, and 40 µg/mL) of EOs combined with *α*-MSH (1 nM) as a co-treatment for a period of 48 h. Subsequently, the cells were lysed using a solution containing 1 M NaOH in 10% DMSO and incubated at a temperature of 90 °C to dissolve the melanin. The relative quantity of melanin was determined by measuring the absorbance at a wavelength of 475 nm utilizing a microplate reader (Multiskan GO, Thermo Fisher, Waltham, MA, USA). Kojic acid (KA, 71 µg/mL), renowned for its skin-lightening properties, was utilized as a positive control.

Cellular tyrosinase activity was evaluated using a DOPA oxidase assay, following the experimental procedure described by Li et al. (2010) [63]. B16/F10 cells were exposed to different concentrations of EOs (10, 20, 30, and 40 µg/mL) in combination with α-MSH (1 nM) for a duration of 48 h. KA served as a positive control at a concentration of 71 µg/mL. The cells were rinsed with PBS and then lysed in a phosphate buffer solution (at pH of 6.8) containing Triton-X/PBS at a ratio of 1% per well (90 µL). After thawing and thorough mixing, L-DOPA solution was added to each well at a concentration of 1%. Subsequently, the mixture was incubated at a temperature of 37 °C for a period lasting 2 h. Finally, tyrosinase activity was quantified using an ELISA kit (Mlbio, Shanghai, China).

### 4.6. Cellular NO, TNF-α, and IL-6 Expression Assay

Nitrite, indicating nitric oxide (NO) synthesis, was quantified using the Griess reaction, with minor modifications to the method outlined by Chou et al. (2018) [13]. RAW 264.7 macrophages were subjected to different doses of EOs (5, 10, 15, and 20 µg/mL) for 0.5 h. After that, they were incubated with LPS at 1 µg/mL for 24 h, with dexamethasone (DEX) at 200 µg/mL utilized as the positive control. Subsequently, 100 µL of cell culture media was mixed with 100 µL of Griess reagent in a 96-well culture plate. Following a 5 min incubation, the optical density was assessed at 540 nm with a microplate reader to ascertain the NO concentration by constructing a standard NaNO_2_ using a serial dilution curve.

For the assessment of interleukin-6 (IL-6) and tumor necrosis factor-*α* (TNF-*α*) levels in the culture supernatants, RAW 264.7 macrophages were treated with different concentrations of EOs (5, 10, 15, and 20 µg/mL) for 0.5 h followed by LPS stimulation (1 µg/mL) for 24 h, with DEX acting as the positive control at a concentration of 200 µg/mL. The concentrations of IL-6 and TNF-*α* were quantified utilizing specialized mice ELISA kits (Mlbio, China) following the manufacturer’s guidelines.

### 4.7. Network Pharmacology

#### 4.7.1. Screening the Active Compounds and Targets of AEO and LEO

The corresponding targets for AEO and LEO were identified using multiple databases, including SuperPred (https://prediction.charite.de/ (accessed on20 May 2024)), Swiss Target Prediction (http://swisstargetprediction.ch/ (accessed on 20 May 2024)), and TargetNet (http://targetnet.scbdd.com (accessed on 20 May 2024)) databases. All target predictions were restricted to the species “Homo sapiens”. Disease-related targets for skin pigmentation and skin inflammation were identified via searches in multiple databases, including OMIM (https://omim.org/ (accessed on 21 May 2024)), DisGeNET (https://www.disgenet.org/ (accessed on 21 May 2024)), and GeneCards (https://www.genecards.org/ (accessed on 21 May 2024)). The keywords “skin whitening”, “skin pigmentation”, and “cosmetology” were used to query targets for skin pigmentation, and the keyword “skin inflammation” was employed for skin inflammation, leading to the identification of relevant disease targets in each respective database. The intersection targets of component and disease were analyzed using jvenn (http://www.bioinformatics.com.cn/ (accessed on 22 May 2024)). Furthermore, all target protein information was standardized through the Uniprot database (http://www.uniprot.org/ (accessed on 8 June 2024)).

#### 4.7.2. Construction of Active Compound–Target Network

The visualization of the compound–target network was executed using Gephi 0.10, while the network topological analysis was performed with the NetworkAnalyzer and Cyto-Hubba plugin in Cytoscape 3.10. In this network, each node signifies an ingredient or a protein target, while a connecting line denotes their relationship. The degree distribution, indicating the fraction of randomly chosen nodes with a particular number of connections, is represented as P(*k*). This calculation follows the methodology described by Wang et al. (2017) [64]. Selection of the compound-target network for the top 5 core components was based on the maximal clique centrality (MCC) value of topological analysis.

#### 4.7.3. Protein–Protein Interaction Network Construction

The component and disease intersection targets were uploaded to the STRING online database (https://string-db.org/ (accessed on 15 June 2024)) to acquire information on the protein–protein interaction (PPI) network. Filtering criteria specified the species as “Homo sapiens” and required a minimum interaction score classified as “medium confidence (0.400)”. In the network diagram, the parameters for each node, such as degree value, betweenness centrality (BC), and closeness centrality (CC), were calculated utilizing the CentiScaPe 2.2 plugin in Cytoscape. For further analysis, target nodes above the median values of degree, betweenness centrality (BC), and clustering coefficient (CC) in the PPI network were considered core targets for subsequent investigations. Finally, based on the filtered core targets, the core PPI network was created and visualized through Gephi 0.10.

#### 4.7.4. GO and KEGG Enrichment Analysis

Gene Ontology (GO) analysis and Kyoto Encyclopedia of Genes and Genomes (KEGG) enrichment were performed utilizing the DAVID online database (https://david.ncifcrf.gov/ (accessed on 8 June 2024)) to investigate the biological functions of the filtered core targets. The select identifier option was set to “OFFICIAL GENE SYMBOL” and the species to “Homo sapiens”. The ten foremost biological processes (BPs), molecular functions (MFs), cellular components (CCs), and the thirty principal pathways were selected, which were visualized and analyzed through an online bioinformatics platform (http://www.bioinformatics.com.cn/ (accessed on 22 May 2024)). The ClueGO and CluePedia plugins in Cytoscape were used to construct a functional cluster network, facilitating further exploration of the multi-scale pharmacological effects of AEO and LEO.

#### 4.7.5. Molecular Docking and Visualization

Protein receptor preparation involves the following steps: removing water, replacing it with hydrogen, designating the protein as a receptor, and saving the structure as a PDBQT protein receptor file using AutoDock 4 software. Similarly, the settings for the compound ligand were configured as follows: optimize the chemical structure of the main active ingredients using Chem 3D 15.0, delete water, add hydrogen, set it as ligand, and export it to a ligand file in PDBQT format using AutoDock 4 software. Molecular docking operations were executed using AutoDock Vina 1.1.2 software, and the outcomes were subsequently visualized using PyMOL 2.3.4 software.

### 4.8. Statistical Analysis

All assays were conducted at least three times, each utilizing different sample preparations. The mean ± standard deviation (SD) was utilized to describe the results, and ANOVA was applied to assess differences among several groups, followed by Tukey’s post hoc test. Graphs were generated utilizing GraphPad Prism 8.0. Statistical significance for all comparisons was determined at *p*-values < 0.05. Principal component analysis (PCA) was employed to visualize the composition difference of EOs, utilizing the STAMP 2.1.3 software. The AEO and LEO composition difference was examined using the permutational multivariate analysis of variance (PerMANOVA) function from the “vegan” package in R, with significance confirmed through 999 permutations.

## 5. Conclusions

This study provides a detailed comparative analysis of the EOs derived from the arils and leaves of *T. grandis*. The chemical analysis revealed a high consistency in the composition of compounds present in both EOs, while the concentrations of these compounds varied. Both AEO and LEO exhibited significant anti-melanogenic, antioxidant, and anti-inflammatory activities. Network pharmacology approaches further supported the therapeutic potential of AEO and LEO, highlighting shared molecular interactions and pathways between the two EOs. Regulating inflammatory cytokines may be one of the critical pathways by which AEO and LEO regulate skin pigmentation. This analysis reinforces the potential utility of AEO and LEO in cosmetic applications, particularly in skin care formulations aimed at reducing pigmentation and inflammation. The leaves of *T. grandis* could be a valuable source of supplementary materials for the production of *T. grandis* aril EO.

## Figures and Tables

**Table 1:** Comparison of EOs from different parts of *T. grandis* in terms of yield and chemicals.

No.	Compounds ^(a)^	Formula	RT ^(b)^	RI ^(c)^	Library RI ^(d)^	AEO	LEO
						Relative Area Percentage (%)	
1	tricyclene	C_10_H_16_	9.28	920	925	0.25 ± 0.02	N/A
2	*α*-pinene	C_10_H_16_	9.74	933	937	39.15 ± 1.23	21.26 ± 0.27
3	*α*-fenchene	C_10_H_16_	10.19	945	950	0.12 ± 0.07	0.19 ± 0.01
4	camphene	C_10_H_16_	10.23	947	952	0.99 ± 0.05	0.29 ± 0.01
5	sabinen	C_10_H_16_	11.16	972	974	0.96 ± 0.05	0.35 ± 0.02
6	*β*-pinene	C_10_H_16_	11.26	975	979	3.10 ± 0.05	1.27 ± 0.07
7	1-octen-3-ol	C_8_H_16_O	11.41	979	981	N/A	1.27 ± 0.02
8	*β*-myrcene	C_10_H_16_	11.85	991	991	3.33 ± 0.17	1.56 ± 0.09
9	3-carene	C_10_H_16_	12.52	1009	1011	3.89 ± 0.16	4.51 ± 0.21
10	p-cymene	C_10_H_14_	13.05	1024	1025	0.76 ± 0.04	0.43 ± 0.03
11	D-limonene	C_10_H_16_	13.25	1029	1029	25.57 ± 0.53	19.04 ± 1.04
12	*β*-ocimene	C_10_H_16_	13.59	1038	1037	0.17 ± 0.01	N/A
13	trans-*β*-ocimene	C_10_H_16_	13.97	1049	1049	0.20 ± 0.01	N/A
14	*α*-terpinolene	C_10_H_16_	15.44	1088	1088	0.38 ± 0.02	0.26 ± 0.01
15	4-terpineol	C_10_H_18_O	18.67	1178	1177	0.35 ± 0.02	0.14 ± 0.01
16	p-cymen-8-ol	C_10_H_14_O	18.93	1185	1183	0.27 ± 0.01	0.14 ± 0.07
17	*α*-terpineol	C_10_H_18_O	19.14	1191	1189	0.30 ± 0.01	0.10 ± 0.01
18	citronellol	C_10_H_20_O	20.46	1228	1228	0.40 ± 0.02	0.10 ± 0.01
19	bornyl acetate	C_12_H_20_O_2_	22.49	1286	1285	0.15 ± 0.01	0.27 ± 0.01
20	*δ*-eIemene	C_15_H_24_	24.40	1341	1338	0.08 ± 0.04	0.14 ± 0.01
21	*α*-terpinyl acetate	C_12_H_20_O_2_	24.79	1352	1350	N/A	0.40 ± 0.01
22	*α*-cubebene	C_15_H_24_	24.83	1353	1351	0.41 ± 0.02	0.62 ± 0.01
23	copaene	C_15_H_24_	25.73	1379	1376	0.19 ± 0.01	0.16 ± 0.01
24	*β*-cubebene	C_15_H_24_	26.20	1393	1389	0.17 ± 0.01	0.32 ± 0.02
25	*β*-elemene	C_15_H_24_	26.25	1394	1391	0.11 ± 0.01	0.19 ± 0.01
26	caryophyllene	C_15_H_24_	27.14	1423	1419	0.29 ± 0.01	0.9 ± 0.02
27	cis-*β*-copaene	C_15_H_24_	27.43	1433	1432	0.83 ± 0.05	1.53 ± 0.07
28	aromandendrene	C_15_H_24_	27.74	1443	1440	0.20 ± 0.01	0.13 ± 0.01
29	trans-*β*-farnesene	C_15_H_24_	28.21	1459	1457	2.05 ± 0.10	3.32 ± 0.03
30	isocadinene	C_15_H_24_	28.75	1478	1477	N/A	0.43 ± 0.01
31	*γ*-muurolene	C_15_H_24_	28.84	1481	1481	1.01 ± 0.05	1.51 ± 0.06
32	germacrene D	C_15_H_24_	28.98	1485	1485	0.64 ± 0.04	2.48 ± 0.08
33	*β*-selinene	C_15_H_24_	29.13	1491	1486	0.06 ± 0.01	0.13 ± 0.01
34	*γ*-amorphene	C_15_H_24_	29.29	1496	1493	0.51 ± 0.03	2.01 ± 0.07
35	viridiflorene	C_15_H_24_	29.37	1499	1496	0.25 ± 0.02	0.52 ± 0.01
36	*α*-muurolene	C_15_H_24_	29.51	1504	1499	1.61 ± 0.08	3.36 ± 0.04
37	*γ*-cadinene	C_15_H_24_	29.89	1519	1513	0.60 ± 0.03	0.81 ± 0.03
38	cis-calamenene	C_15_H_24_	30.14	1528	1523	N/A	6.25 ± 0.16
39	*δ*-cadinene	C_15_H_24_	30.15	1529	1524	3.42 ± 1.24	7.95 ± 0.08
40	germacrene B	C_15_H_24_	31.06	1563	1557	N/A	0.21 ± 0.01
41	trans-nerolidol	C_15_H_26_O	31.14	1566	1564	N/A	0.25 ± 0.02
42	(−)-globulol	C_15_H_26_O	31.35	1574	1576	N/A	0.10 ± 0.01
43	spathulenol	C_15_H_26_O	31.59	1583	1580	0.27 ± 0.02	0.57 ± 0.01
44	gleenol	C_15_H_26_O	31.76	1590	1586	0.99 ± 0.05	3.19 ± 0.05
45	epicubenol	C_15_H_26_O	32.86	1634	1627	0.54 ± 0.03	2.50 ± 0.04
46	*δ*-cadinol	C_15_H_26_O	33.28	1652	1645	0.88 ± 0.03	3.94 ± 0.04
47	*α*-cadinol	C_15_H_26_O	33.49	1661	1653	0.13 ± 0.01	0.71 ± 0.03
48	cis-10-hydroxycalamene	C_15_H_26_O	33.58	1665	1666	0.46 ± 0.01	0.40 ± 0.01
49	trans-10-hydroxycalamenene	C_15_H_26_O	33.79	1674	1676	0.81 ± 0.03	0.66 ± 0.01
50	trans-farnesol	C_15_H_26_O	34.96	1724	1713	N/A	0.78 ± 0.11
	Total identified					96.86 ± 1.54	97.65 ± 0.05
	Monoterpene hydrocarbons					78.43 ± 1.09	48.84 ± 0.72
	Oxygenated monoterpenes					1.17 ± 0.04	2.32 ± 0.10
	Sesquiterpene hydrocarbons					12.42 ± 1.24	32.96 ± 0.67
	Oxygenated sesquiterpenes					4.08 ± 0.15	13.10 ± 0.20
	Yield of TGEO (*v*/*w*)					2.04	0.49

^(a)^ Identified chemical compounds according to the data of GC-MS. ^(b)^ Retention time of identified chemical compound. ^(c)^ Retention index calculated from n-alkanes (C8–C40) on the same silica capillary column. ^(d)^ Reference retention index in NIST 17 database. Note: N/A indicates that the compound was not detected.

**Table 2:** Molecular docking results of main target and active compounds.

Bioactive Compounds		Binding Energies (kcal/mol)						
Name	Pubchem ID	TNF	STAT3	EGFR	CASP3	HIF1A	BCL2	NFKB1
cis-*β*-copaene	87529	−9.6	−7.3	−7.9	−6.5	−5.7	−7.0	*NA*
bornyl acetate	93009	−7.1	−6.1	−6.2	−5.8	−5.0	−6.1	−5.4
tricyclene	79035	−6.8	−5.6	−5.1	−5.6	−5.4	*NA*	−5.5
p-cymen-8-ol	14529	−7.6	−5.9	−6.7	−5.9	−5.8	−6.1	−5.3
p-cymene	7463	−7.6	−5.8	−6.5	−5.9	−5.9	*NA*	−5.3
1-octen-3-ol	18827	−5.5	−5.7	−5.9	−5.5	*NA*	−5.7	−5.0
(−)-globulol	12304985	−9.1	−7.8	−8.1	−6.6	*NA*	−6.8	*NA*
*β*-eIemene	6918391	−8.3	−6.4	−7.3	*NA*	−5.6	−6.4	−5.6
*α*-terpinyl acetate	111037	−8.2	−6.0	−6.8	*NA*	*NA*	−6.0	−5.8
*α*-pinene	6654	−6.6	−5.6	−5.3	−5.9	−6.0	−5.7	−5.1
D-limonene	440917	−7.3	−5.3	−6.3	−5.3	−5.7	−5.6	−5.2

Note: *N/A* indicates that the protein target is not docked with the compound.

## Data Availability

Data are contained within the article and Supplementary Materials.

## Supplementary Materials

The following supporting information can be downloaded at: https://www.mdpi.com/article/10.3390/plants13182640/s1, Table S1: Putative targets of EOs for the treatment of skin pigmentation and inflammation. Table S2: Topology analysis results of compound-target interaction network. Table S3: Bioinformatic enrichment of AEO in skin pigmentation. Table S4: Bioinformatic enrichment of LEO in skin pigmentation. Table S5: Bioinformatic enrichment of LEO in skin inflammation. Table S6: Bioinformatic enrichment of LEO in skin inflammation. Figure S1: Venn diagram showing the unique and shared differential components from the samples of *Torreya grandis* aril (AEO) and leaves (LEO). Figure S2: The Volcano plot illustrates significant up- or down-regulated components between samples of *Torreya grandis* aril (AEO) and leaves (LEO). Figure S3: Effect of AEO and LEO on the cell viability in *α*-MSH-stimulated B16 cells. Figure S4: Effect of AEO and LEO on the cell viability in LPS-stimulated RAW 264.7 macrophages. Figure S5: The potential targets of active compounds in AEO and LEO intersect with disease-related targets. Figure S6: Degree distribution and the betweenness centrality of the compound-target interaction network. Figure S7: Bar chart of GO enrichment analysis and bubble chart of KEGG enrichment analysis.
