# Supplementary material for: Comparative Analysis of Chemical Profiles and Biological Activities of Essential Oils Derived from Torreya grandis Arils and Leaves: In Vitro and In Silico Studies
Source: Plants (Basel). 2024 Sep 21;13(18):2640. doi: 10.3390/plants13182640 (PMC11434864; doi:10.3390/plants13182640)
Supplement: Supplementary file 1 [file plants-13-02640-s001.zip › Supplementary Figures.pdf]

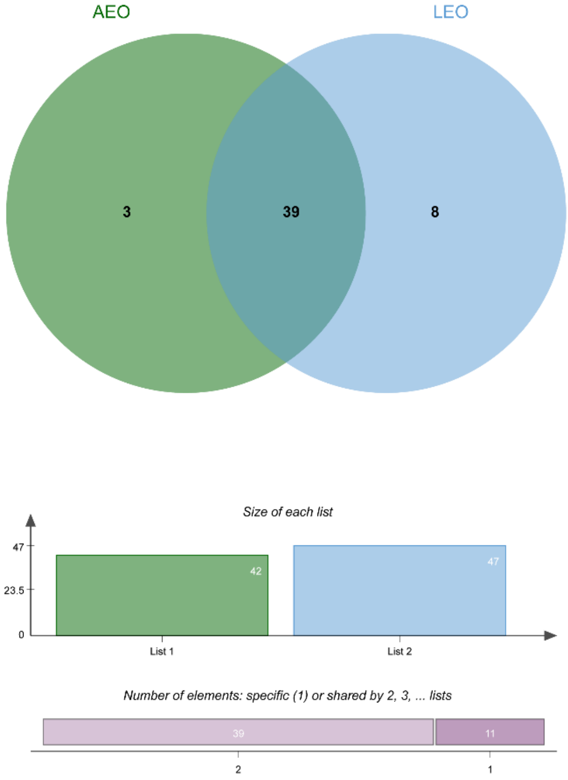

Figure S1. Venn diagram showing the unique and shared differential components from the samples of *Torreya grandis* aril (AEO) and leaves (LEO).

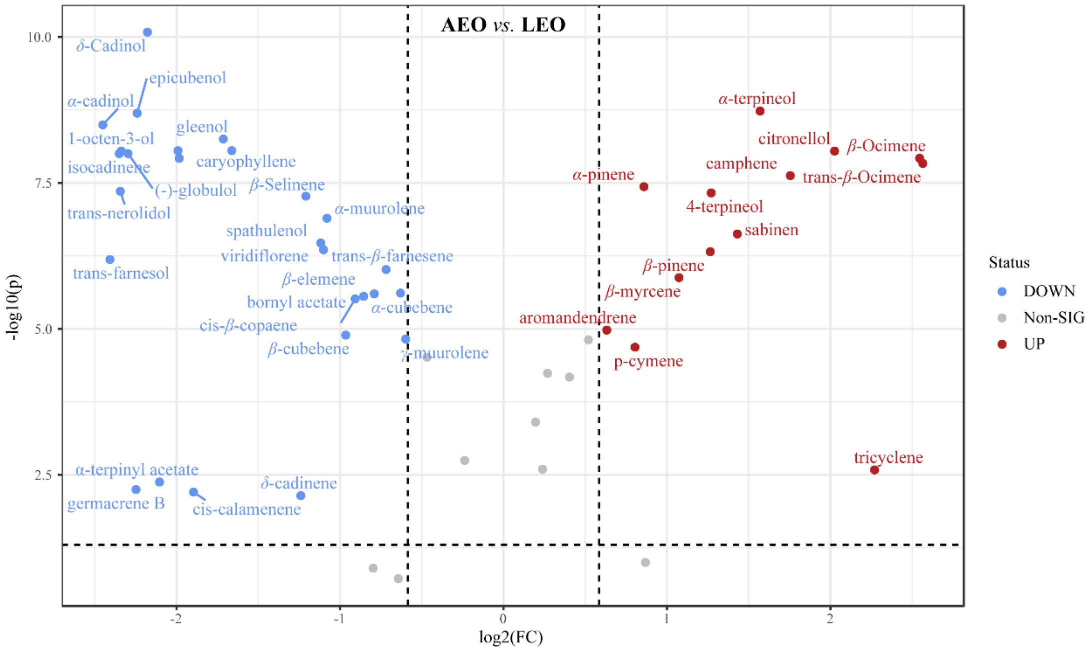

Figure S2. The Volcano plot illustrates significant (p <0.05, FC >1.5) up- or down-regulated components between samples of *Torreya grandis* aril (AEO) and leaves (LEO).

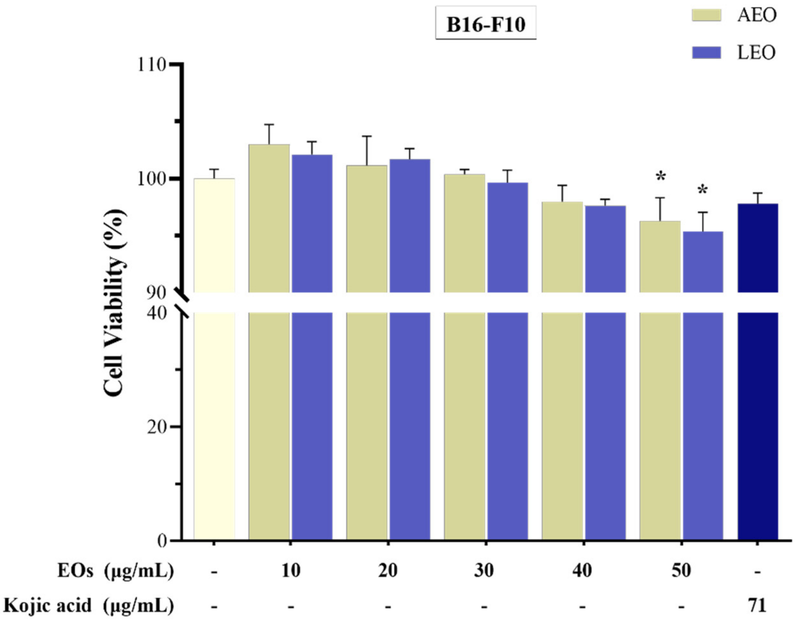

Figure S3. Effect of AEO and LEO on the cell viability in α-MSH-stimulated B16 cells. The data are means ± S.D. ($n = 3$). ∗ indicates a significant difference ($p < 0.05$) compared with the α-MSH-treated group.

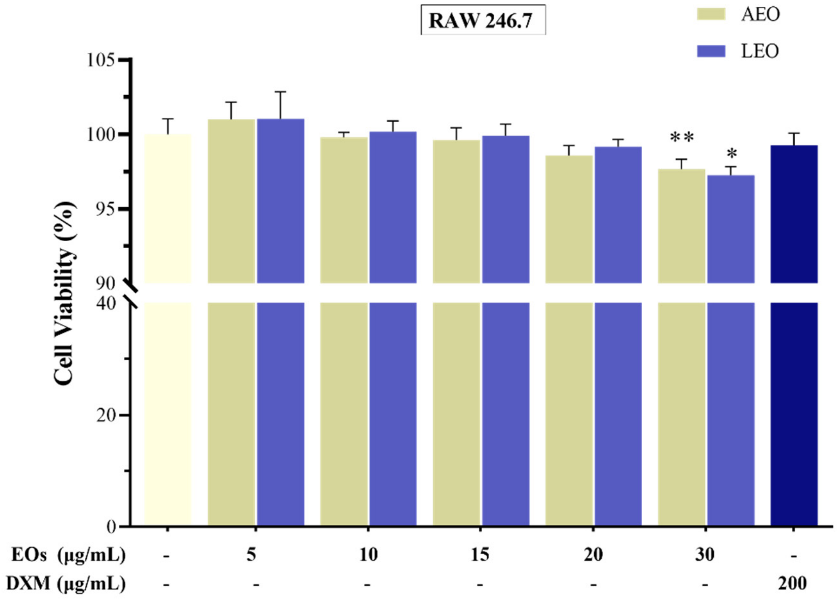

Figure S4. Effect of AEO and LEO on the cell viability in LPS-stimulated RAW 264.7 macrophages. The data are means ± S.D. ($n = 3$). ∗ indicates a significant difference ($p < 0.05$) and ∗∗ indicates a highly significant difference ($p < 0.01$) compared with the LPS-treated group.

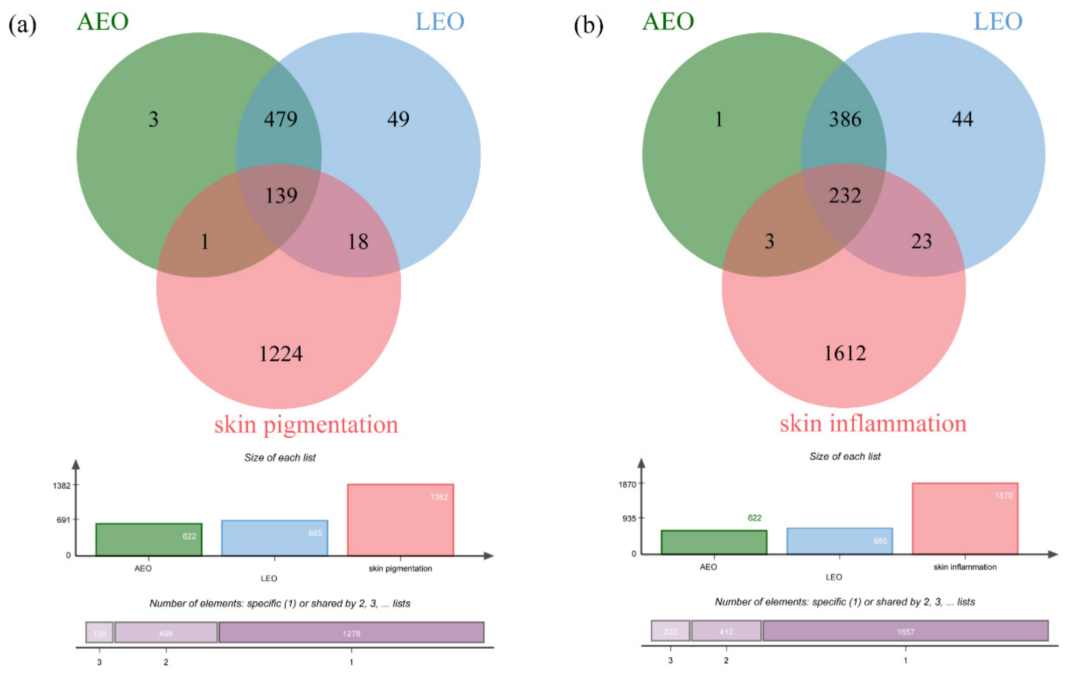

Figure S5. The potential targets of active compounds in AEO and LEO intersect with disease-related targets. Active compounds intersect with **(a)** skin pigmentation-related targets and **(b)** skin inflammation-related targets.

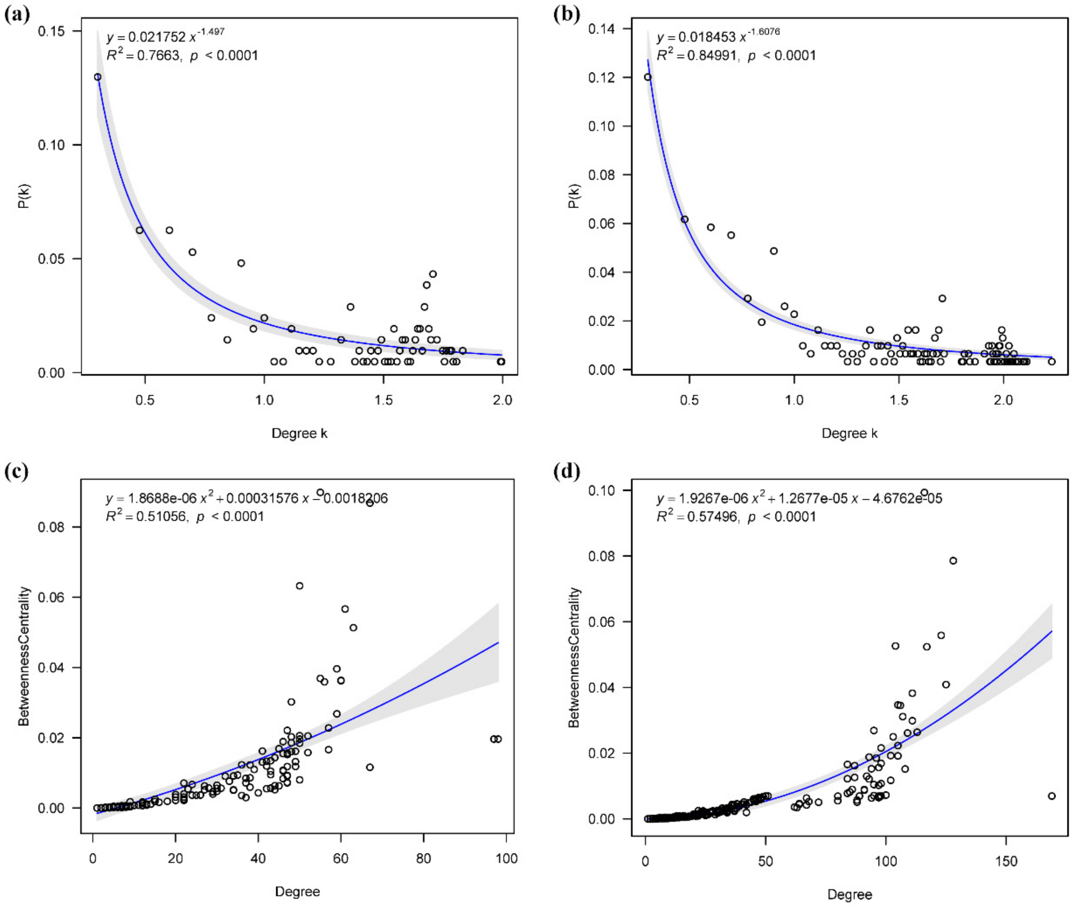

Figure S6. Degree distribution and the betweenness centrality of the compound-target interaction network. The degree distribution of **(a)** EOs-skin pigmentation network and **(b)** skin inflammation network. k represents degree values, and that P(k) indicates degree distribution. The distribution of betweenness centrality versus unweighted degree in **(c)** EOs-skin pigmentation network and **(d)** betweenness centrality distribution of EOs- skin inflammation network.

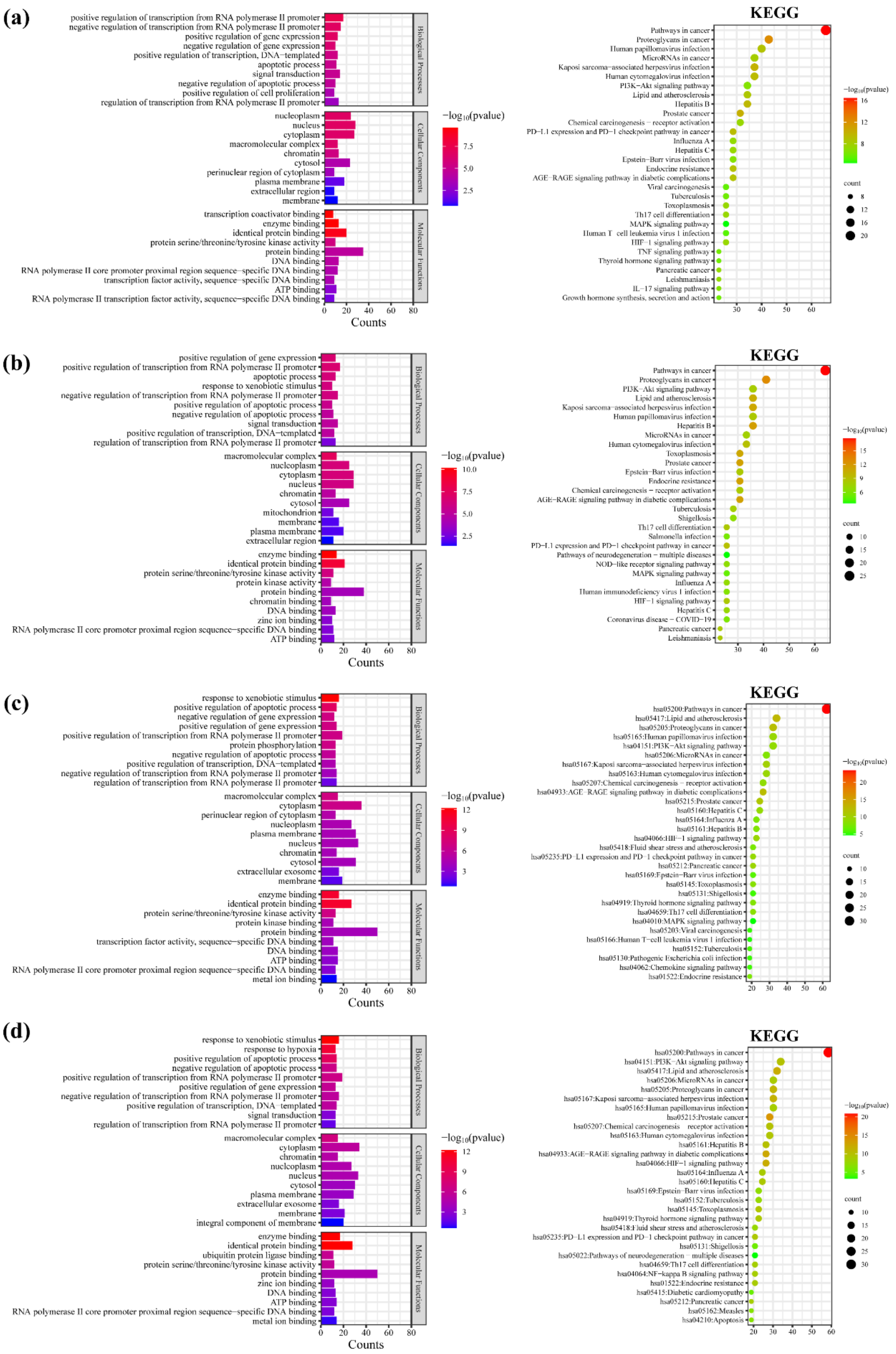

Figure S7. Bar chart of GO enrichment analysis and Bubble chart of KEGG enrichment analysis. Top 10 most significant ($p_{.adjust}$ < 0.05) results of GO enrichment analysis in **(a)** AEO in treating skin pigmentation and **(b)** LEO in treating skin pigmentation. Top 30 most significant ($p_{.adjust}$ < 0.05)

signal pathways of KEGG enrichment analysis in **(c)** AEO in treating skin inflammation and **(d)** LEO in treating skin inflammation.

,
